# Should reference values for ventricular volumes and mass of children be indexed for body surface area, height or weight considering gender differences?

**DOI:** 10.1186/1532-429X-11-S1-O97

**Published:** 2009-01-28

**Authors:** Samir Sarikouch, Titus Kuehne, Matthias Gutberlet, Philipp Beerbaum

**Affiliations:** 1 Department of Heart-, Thoracic-, Transplantation- and Vascular Surgery, Hannover Medical School, Germany; 2 Unit of Cardiovascular Imaging – Congenital Heart Diseases, Deutsches Herzzentrum, Berlin, Germany; 3 Department of Radiology, Heart Centre, Leipzig, Germany; 4 Division of Imaging Sciences, King's College, Guy's & St Thomas' Hospital, London, UK

**Keywords:** Stroke Volume, Body Surface Area, Ventricular Volume, Significant Gender Difference, Main Pulmonary Artery

## Background

Medical decision making in congenital and acquired heart disease is based increasingly on quantitative MRI assessment of ventricular volumes and myocardial mass. However, for children in different age groups, there is only very limited reference data available, yet. The objective of this study was therefore to provide statistically robust reference data for ventricular volumes and mass for healthy children.

## Methods

A total of 114 healthy children and adolescents, uniformly distributed, were examined in a standard 1.5 Tesla scanner in breathhold-technique using steady-state free precession and phase-contrast sequences according to a standardized pediatric CMR protocol. Transversal acquisition, 5–6 mm slice-thickness, no gap, 25–35 phases, resolution 2.0–2.5 × 1.5–1.8 mm^2^. Semiautomatic volumetric analysis as well as analysis of stroke volumes in the main pulmonary artery and the ascending aorta were performed by one observer to minimize observer error.

## Results

**Figure 1:**
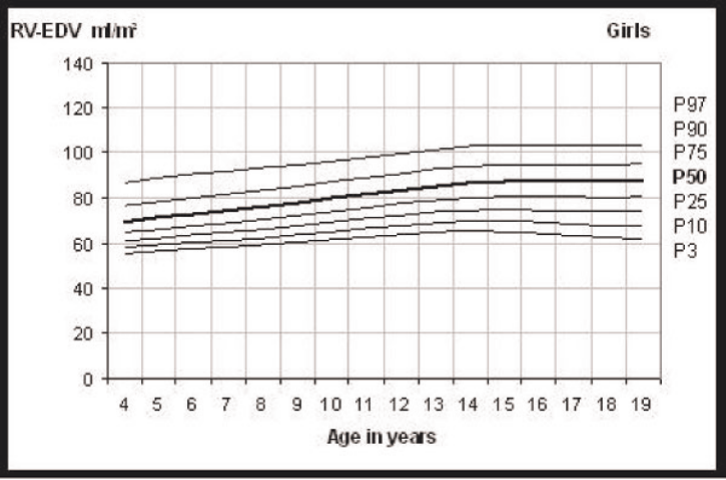
Figure 1

114 children and adolescents were examined, age ranged between 4 and 20 years (25. percentile 9,2 yrs, 50. percentile11,9 yrs, 75. percentile 16,1 yrs). Reference centile curves were constructed using the lambda-mu-sigma (LMS) method for left and right enddiastolic and-endsystolic volumes, stroke volume, ejection fraction and ventricular mass. Figure 1.

With increasing age there was a steady increase in left and right ventricular enddiastolic volumes indexed for body surface area, as the most common form of indexing in MRI, from 4 up to 14 years where the volumes reached a plateau. Ventricular volumes and masses indexed to the body surface area were in general 10% higher in boys than in girls. This statistically significant gender difference showed to be the same when indexing for height and disappeared after relating ventricular volume to weight. 2.5 ml/kg resulted in the enddiastolic volume of the ventricles and 1 ml/kg in the endsystolic volume.

## Conclusion

Data for ventricular volumes and mass for children are provided which can serve as a reference tool for the assessment of pathologic changes in congenital and acquired heart disease. Indexing of ventricular volumes to body weight instead of body surface area elimates the gender difference in children and should therefore be preferred.

